# Organized Scientific Diaspora and Its Contributions to Science Diplomacy in Emerging Economies: The Case of Latin America and the Caribbean

**DOI:** 10.3389/frma.2022.893593

**Published:** 2022-05-19

**Authors:** Luisa F. Echeverría-King, Reina Camacho Toro, Pedro Figueroa, Laura A. Galvis, Alejandra González, Verónica Rossana Suárez, Ivonne Torres Atencio, Claudia Natalie Widmaier Müller

**Affiliations:** ^1^Corporación Universitaria del Caribe, Sincelejo, Colombia; ^2^UMR7585 Laboratoire Physique Nucléaire et Hautes Energies (LPNHE), Paris, France; ^3^DiploCientifica, Santiago, Chile; ^4^Australian Regenerative Medicine Institute (ARMI) Department, Clayton, VIC, Australia; ^5^Departamento de Emprendimiento e Innovación, Universidad de La Sabana, Chía, Colombia; ^6^Pharmacology Department, University of Panama, Panama City, Panama; ^7^Organization for Women in Science for the Developing World, Trieste, Italy

**Keywords:** scientific diaspora, science diplomacy, emerging economies, research policy, Latin America, diaspora networks, international cooperation

## Abstract

The current knowledge society has driven an unprecedented mobility of people, especially scientists, from emerging economies to developed countries. This mobility can allow the development of human talent and the access to first class infrastructure and resources, but it can also mean a loss for emerging economies due to the phenomenon of brain drain. To counteract this situation, some countries in Latin America and the Caribbean have developed models for the articulation of their scientific diaspora in projects and programs, with the aim of exchanging knowledge and capitalizing on human and technical resources to advance science, technology and innovation systems. Likewise, science diplomacy has become a tool for interlinking the work of various actors in order to advance the solution of national, transnational or global problems through scientific advice. Scientific diasporas are vital in new structures of cooperation, enabling them to innovate and solve problems jointly, advising their countries of origin and articulating policies and programs. This research seeks to analyze the interactions and initiatives identified between the organized scientific diaspora from Latin America and the Caribbean and their countries of origin in relation to science diplomacy processes, providing recommendations and proposals for public policy to improve the interaction between the diaspora and the governments of their countries of origin. Results show that diaspora organizations from Latin America and the Caribbean engage with governmental and non-state actors and are active science diplomacy stakeholders promoting the scientific developments of their country or their researchers, as well as enabling access to research resources creating alliances for scientific, institutional and academic collaborations. In the cases studied, these efforts are planned and executed by the diaspora without responding to any science diplomacy strategy of the country. Policies and programs are needed to effectively link the scientific diaspora organizations to the interests of the countries.

## Introduction

The concept of diaspora comes from the ancient Greek and refers to dispersing seeds (Plaza, 2013); currently it refers to the relocation of a group of people from their place of origin to a new country, creating a connection not only with their homeland, but also with the host country. Diasporas also have a group consciousness, creating their own identity in relation to their country of origin and members of other regions or communities (Fernández, 2008). Diasporas build bridges between societies and create mutually beneficial transnational communities in terms of development for host and home countries. Strategic partnerships between States, international organizations, civil society, and industry provide a framework for diaspora engagement and empowerment by sharing and transferring their resources. The role of diasporas in the human, social, and economic development of countries, poverty reduction, reconstruction and growth is gaining considerable interest globally. Integrating diasporas into local development projects that represent a real added value for national economies is at the heart of migration management, as it establishes effective cooperation between countries of origin and destination to support their diasporas in contributing to development (IOM, 2007).

The mobility of highly-qualified workers from emerging economies to developed countries can foster human talent and access to first class infrastructure and resources, but it can also mean a loss for emerging economies due to the phenomenon of brain drain. However, the expatriate skilled population can be also considered as a potential asset instead of a loss. In particular, when referring to scientists and engineers abroad. If the countries of origin can use the scientific diaspora as a resource then the gains can be substantial. Science diplomacy (SD) strategies in developing countries often aim to articulate the scientific diaspora to capitalize on the human and technical resources, and advance national science, technology and innovation (STI) systems, which enables the paradigm shift toward the circulation of knowledge. Through a quantitative survey to 10 diaspora organizations and three qualitative focal group studies, we unveil how the organized scientific diaspora from Latin America and the Caribbean (LAC) currently understands and contributes to SD processes. Based on these results we propose public policy recommendations to improve the interaction between the diaspora and the governments of their countries of origin.

This article is organized as follows: first, conceptual approaches regarding the scientific diaspora and SD will be introduced. Next, the situation of the Latin American and Caribbean scientific diaspora will be addressed, including some examples of programs intended to engage the scientific diaspora with their countries of origin. The methodology of the study will then be presented and both quantitative and qualitative results will subsequently be reported. The discussion section will be followed by recommendations for scientific diaspora organizations and governments and public policy implications. The article ends with a section of conclusions.

## Scientific Diaspora: Conceptual Approaches

There are different types of diaspora, which respond to different reasons for displacement. Within the framework of the current knowledge society, global competition for talent has increased dramatically, causing the mobilization of professionals in search of a better future, especially from emerging economies to industrialized countries. The International Organization for Migration Migration Policy Institute (2012), highlight the importance of creating an enabling environment for diaspora engagement through the creation of “diaspora and development” programs and the implementation of a commensurate regulatory framework that promotes benefits and obligations focused on a highly mobile population. This article will focus on the diaspora that migrates for scientific purposes: to advance their scientific career or to work in sectors requiring specialized, scientific skills. Gëdeshi and King (2019) describe as scientific diaspora a country's scientific talent moved abroad, including those who obtain high level training abroad. From a transnational approach, the scientific diaspora refers to highly qualified migrants who not only acquire knowledge in the host country, but also contribute to their homeland, becoming agents of development facilitating knowledge, connections and technology transfer (Tejada, 2007). For the purposes of this research, we will use this transnational approach of the scientific diaspora to understand the possible connection between this phenomenon and SD processes.

According to Shin and Moon (2018) there are three approaches to the migration of high-skilled nationals or what the authors refer to as the approaches to *brain power*: First, the *brain drain* approach, which refers to the negative impact of migration in the scientific landscape of a country and the loss of talent that results from emigration. In traditional schemes, the diaspora has been considered a *brain drain* for developing countries. Second, the *brain circulation* approach, common in less developed countries, recognizes a potential positive impact of migration only when migration is followed by the return to the home country. And third, the *brain linkage* approach, which refers to the power of the home-host interactions in which emigrants decide to engage, even when they do not return to their home country. There are a number of determinants that may support or hinder the effectiveness of a *brain linkage* approach, such as information about institutional initiatives, a stable political landscape and adequate STI infrastructure in the home country (Tejada et al., 2013).

Policymakers and public policy play an important role in enabling and fostering cooperation and engagement of the scientific diaspora with their countries of origin. According to Martinez Pizzaro (2005), the brain drain is a problem that should be addressed through public policy schemes for the following reasons: (i) The scarce initiatives to encourage the return of qualified emigrants prevent the internalization of strategies in the field of STI, and the benefit of their valuable experiences; (ii) The existing networks have been sporadic, and have not had sustained governmental support over time, despite having been conceived as unique meeting spaces between scientific diasporas and local communities; (iii) The organized diaspora finds acceptance among well-organized migrant communities, but it is not directly embraced in the context of STI.

Scientific diaspora may become organized through diaspora networks. These networks refer to a map of actors where diaspora participates with key actors both in the home and host country, such as universities, companies, research centers, international cooperation agencies, among others. Organized scientific diaspora networks facilitate the interaction and knowledge transfer between the country of origin and the host country. These networks emerged due to the interest of individuals abroad to gather and take advantage of a shared identity and culture, to contribute to their home country and sometimes thanks to explicit national policies and programs that promote linkages with the scientific diaspora.

The digital era has supported the consolidation of these scientific diaspora networks, establishing meaningful virtual connections with various stakeholders in their home and host countries to advance joint projects and activities (Grossman, 2010), in particular for the development of STI. Organized scientific diaspora networks may boost innovation by supporting and building commercial bridges, facilitating access to products and services developed abroad and enabling value-generating investments without having to return home (Epstein and Heizler, 2016). Thus, from a transnational and brain linkage approach, scientific diaspora becomes a key actor for science and innovation diplomacy schemes (Echeverría King et al, 2021), especially for emerging economies like those in LAC with a large population of expatriates not intending to return to their home countries.

## Science Diplomacy

SD is experiencing a time of excitement in LAC. Initiatives on SD across this region tend to grow on a sound basis (Gual, 2020). In spite of that, the concept of SD remains widely unknown in LAC. This is mainly because the term SD is relatively new. Even though the interactions between researchers and diplomats date from long ago, this concept received its first taxonomy in 2010, to encompass the intersection between the actions on Science, Technology and Innovation, and those of the international community (The Royal Society, 2010).

The wider literature defining this concept confirms that there is no such thing as a one-size-fits-all SD approach (Rungius et al., 2018). Since 2010, many efforts have attempted to provide a definition of SD. According to Turekian (2018) SD supports the development of science and knowledge exchanges that go beyond the generation of scientific achievements. Epping (2020) explains that SD considers science to be a vehicle to foreign policy goals by addressing the pre-political room in the sense of operating as a depoliticizing element and unfolding impact in different ways than traditional diplomacy.

Although this region has no common definition for SD, three specific dimensions are identified in most of these countries, which help shed some light about how this concept is understood in LAC. For this purpose, the “strategic purposes approach” from Flink and Schreiterer (2010) distinguishes three types of actions undertaken by State stakeholders to enact SD. First, “Access” refers to actions aiming to support the competitiveness of a national STI ecosystem at global scale. Second, “Promotion” seeks to improve the reputation and attractiveness of a country by highlighting its performance on STI. Third, “Influence” includes the actions of a nation designed to engage the foreign policy-makers and the public opinion by taking STI as an entry point.

A more utilitarian approach than the one here above sorts out the actions on SD into three categories. First, the actions to advance a country's national needs; second, the actions to address cross-border interests; and third, the actions primarily designed to meet global needs and challenges (Gluckman et al., 2017).

SD in emerging economies can have different approaches and facets. It can serve for capacity building, development of human talent and fostering of knowledge and technology transfer (Arunachalam et al., 2017). Also, SD has supported emerging economies to establish meaningful partnerships to work toward the Sustainable Development Goals of the 2030 Agenda (Thompson, 2018; Echeverría et al., 2020). It also fosters the search for solutions to regional or transnational health challenges (Jarquin-Solis and Mauduit, 2021), as seen during the Covid-19 pandemic. The alliances promoted by SD have helped to leverage South-South relations, such as between Brazil, Russia, India, China and South Africa (the BRICS countries); sharing resources and infrastructure in order to solve common problems (Bonilla et al., 2021). Through the exchange of experts and the installation of capacities in emerging countries, SD also facilitates a balance of power and alleviates asymmetries between the North and the Global South (Hornsby and Parshotam, 2018), reducing bad practices such as neo colonialism in international relations and science cooperation. SD also promotes the development of resources and conditions for the return of the scientific diaspora to the country of origin (IOM, 2007).

## The Scientific Diaspora and Science Diplomacy in Latin America and the Caribbean

As Latin American governments established national STI systems (Organización Internacional para las Migraciones, 2007), the need for human capital strengthening policies became apparent. This scenario has created better conditions for the skilled diaspora to be considered an important actor within the innovation and scientific development system. In this sense, several Latin American governments are making efforts to encourage the return of skilled migrants or to contribute to networks between professionals and scientists living abroad and those in the country.

In Table 1, we present different public initiatives from LAC that demonstrate the increasing recognition of the scientific diaspora as a local human resource through the development of policies and programs focused on generating spaces for exchange or stimuli for return. For example, Colombia had a particular case with the linkage of the organized scientific diaspora through the Caldas Network that ended between 2000 and 2002. With COLCIENCIAS as coordinator, the network linked student and professional associations abroad, which facilitated the start of the network and proved to be one of its success factors (Chaparro et al., 2004). For its part, the Chilean government created DICOEX in 2000, whose objective is to strengthen relations with Chilean professionals living abroad and to promote cooperation and knowledge transfer between them and the country (IOM, 2007). DICOEX has a database of Chilean organizations abroad that are easily accessible through its website, helps fund existing associations abroad, and provides information to expatriates (Ministerio de Relaciones Exteriores de Chile (MRREE), 2021).

**Table 1:** Examples of programs, laws or public policies regarding the highly qualified diaspora in Latin America and the Caribbean from 1990 to 2021.

**Country**	**Program/law/public policy**	**Year**	**Description**	**References**
Argentina	Crear – Revinculación de científicos, técnicos y profesionales Argentinos	1999-?	Promote communication between scientists abroad and the local scientific community.	Tigau, 2010
	Programa Nacional para la Vinculación con Científicos y Técnicos Argentinos en el Exterior (PROCITEXT)	1990-1999	Coordinate institutional efforts, design initiatives to benefit from the capacity of emigrated researchers, facilitate the return of emigrated researchers who wish to return, and promote linkages with those who remain abroad.”	Lujan Leiva, 2005
	Raíces (Red de Argentinos/as Investigadores/as y Científicos/as en el Exterior)	2003- present	Promote the return, develop linkage policies, promote participation in the construction of the national science, technology and innovation policy of Argentine researchers living abroad. Promote international training and development opportunities for scientists that can be incorporated in the country.	Ministerio de Ciencia Tecnología e Innovación, 2021
Brazil	Actions to Benefit Brazilians Abroad' Action Plan	2011-2012	Organize activities with the diaspora on consular services, politics, education, social security, labor, culture, communication, economy, science and technology.	Ministério das Relações Exteriores, 2017
Caribbean	Tratado de Chaguaramas (revisado)	2001	Allow free movement of CARICOM members through the region.	Comunidad y Mercado Común del Caribe, 1973
	CARICOM-Caribbean Conference on the Diaspora: A 2020 Vision	2007	Identify how to create close links with the diaspora, its resources and knowledge to the advantage of the region.	Minto-Coy, 2009
Chile	DICOEX	2000- present	Promote the inclusion of Chileans abroad in the country's activities, defend their human rights, strengthen their ties and identity with Chile, encourage network formation to promote Chilean talents, train community leaders, inform on public policies that may concern them, study international migration issues, propose the components of the migration agenda and coordinate the participation of the Foreign Ministry in international forums on migration.	Ministerio de Relaciones Exteriores de Chile (MRREE), 2021
Colombia	Colombia Nos Une	2003 - present	Link Colombians abroad and make them subjects of public policies, strengthen the community abroad, accompany the return to the country, identify and establish contact with distinguished expatriates, offer services and benefits that contribute to raising the quality of life of Colombians abroad. According to law 2136, facilitate the execution of cooperation projects of the national diaspora with business, cultural, academic and research projects.	Cancillería de Colombia, 2022a
	Plan de Retorno Positivo	2009- 2011	Support the return of nationals abroad with economic and social opportunities that contribute to the country's development.	Cancillería de Colombia, 2022b
	Es tiempo de volver	2014	Incorporate Colombian doctors residing abroad through postdoctoral fellowships in universities, research centers, technological development centers and companies.	Ministerio de Ciencia y Tecnología de Colombia, 2014
	Ley 1565 del 31 de julio de 2012	2012 - present	Create financial incentives to promote the return of Colombians, and provide support to those Colombians who voluntarily wish to return to the country.	Cancillería de Colombia, 2022c
	Red Caldas	1991-2002	“Integrate Colombian researchers abroad to the national scientific community and to the activities of the National Science and Technology System.”	Chaparro et al., 2004
	Ley 2136 de 2021	2021	Diaspora integration policies. Promotion of diaspora networks through international cooperation mechanisms to improve the quality of research. Guide for Colombia Nos Une	Congreso de la República Colombiana, 2021
Ecuador	Programa Prometeo	2011-2017	Strengthen the country's academic and research capacity by incorporating outstanding scientists, and Ecuadorians living abroad, into the Ecuadorian academy and research centers.	Chiriboga Salazar, 2019
	“Ecuador Saludable, Vuelvo por ti”	2012	Recruit Ecuadorian and foreign health professionals abroad, prioritizing highly trained specialists and sub-specialists to cover the requirements in critical areas and undersupplied regions of Ecuador.	Ministerio de Salud Pública, 2022a,b
	Plan Retorno Educación	2013-?	Recruit professionals in education or other areas from Ecuadorians living abroad.	Ministerio de Educación Ecuador, 2022
Mexico	Red Global MX	2005 - present	Organizes highly qualified Mexicans who live outside the country and are interested in promoting the development and good image of Mexico.	Instituto de los Mexicanos en el Exterior, 2018
Panama	Programa de repatriación de talento	2006-?	Repatriation of internationally recognized Panamanian scientists.	Aguilar, 2022; SENACYT, 2012
Peru	Ley del Retorno (Ley N° 30001)	2013-2016	Facilitate the economic and social reintegration of Peruvian migrants and their families, including the recognition of studies carried out abroad, access to social programs and subsidies. Regarding skilled migration, support from the National Council for Science, Technology and Technological Innovation (Concytec) to scientists and researchers who take advantage of it.	Congreso de la República Peruana, 2013; CONCYTEC, 2020
	Encuentro Cientifico Internacional	1993 - present	Exchange experiences and knowledge, establish cooperative relationships between research centers and institutions in Peru and abroad.	Gual, 2020; ECI, 2022
Suriname	Diaspora Institute Suriname	2017	Organize and structure cooperation with the diaspora worldwide to connect as many diaspora as possible with local projects, people and initiatives.	Diaspora Instituut Suriname, 2022
Uruguay	Programa de Vinculación con Uruguayos Altamente Calificados residentes en el Exterior	2001-2005	Support Uruguayan professionals abroad as qualified human resources for collaboration and contribution to the country's development.	Presidencia de la República Oriental del Uruguay and Secretaría de Prensa y Difusión, 2001
	Departamento 20 (Dirección de Servicios Consulares y de Vinculación con los Uruguayos en el Exterior)	2005- present	Form Advisory Councils in countries with a higher concentration of emigrants and diplomatic representation, create a voluntary consular registry of emigrants and increase the efficiency of the Foreign Service.	Taks, 2006
	Programa de Circulación de Uruguayos Altamente Calificados (CUAC)	2005- present	Link highly qualified Uruguayans living abroad with the country's institutions through this network of academics, business persons, artists and cultural agents.	Ministerio de Relaciones Exteriores de Chile (MRREE), 2021
Venezuela	Talento Venezolano (TALVEN)	1994-?	Link Venezuelans residing abroad, who have studied with the financial support of the State, to their peers in Venezuela, and sponsor the return of Venezuelan scientists to teach in local institutions.	Guellec and Cervantes, 2002

In the case of Argentina, it has a long history of skilled migration. The governmental program “Argentine Network of Researchers and Scientists Abroad” (MINCYT, RAICES, 2021), created by Law N° 26.421, is under the Ministry of Science and Technology (Secretariat of Planning and Policies in Science, Technology, and Innovation). The initiative develops strategies to strengthen the country's scientific and technological capacities through links with the scientific diaspora helping them to create networks and with the local STI system.

In Uruguay, the “Programme for the Circulation of Highly Qualified Uruguayans (CUAC)” has been in place for several years. The Advisory Councils and the “Department 20” within the orbit of the General Directorate of Consular Affairs and Liaison of the Ministry of Foreign Affairs, currently focuses on the term “diaspora of Uruguayans abroad” (although the decree creating Department 20 is still in force). In 2018, an important pilot experience called “active citizenship for development” was carried out. The meeting brought together members of the diaspora interested in contributing their knowledge to Uruguay and deepening their relationship with the country at the scientific, technological, and business levels; with priority in the areas of biotechnology and pharmaceuticals, information technologies, energy, food, forestry, and the country's scientific and technological development (Uruguay Presidencia, 2018).

The integration between highly qualified diaspora networks across Latin America has also been explored more recently. The research and development program “Creation of incubators of knowledge diasporas for Latin America” (CIDESAL), financed by the European Union, sought to establish links and public policies for cooperation with professionals living abroad (Meyer, 2015). The initiative involved the French Institute of Research for Development (IRD), the Centro Redes of Argentina, Colombia Nos Une of Colombia, the Faculty of Social Sciences of the University of the Republic of Uruguay and the Polo Mercosur Foundation.

In addition, the European Union Global Diaspora Facility (Diaspora for Development, 2021) pilot project is an initiative implemented by the International Center for Migration Policy Development (ICMPD) that seeks to consolidate efforts on diaspora engagement in development by encouraging participation and creating a laboratory of innovative ideas and policies, based on needs and priorities. The European Union Global Diaspora Facility created the Diaspora Engagement Map using data from the United Nations (UN) and the World Bank. This map allows the visualization of the Latin American diaspora by country, providing graphs, information on policies and institutions, as well as information on the various projects involving diasporas in each area.

Despite the initiatives mentioned in Table 1 and discussed in the text, diaspora integration policies in LAC are isolated and independent events, of short duration, and where the different actors of society that could benefit from the diaspora are not integrated. There is predominantly unidirectional communication from the government to the diaspora, with few institutional spaces to take into account the experiences of the diaspora in the design of public policies on science and technology. Additionally, a large part of public policies are oriented toward individuals and not necessarily toward the organized diaspora (López-Vergés et al., 2021). In addition, science still plays a secondary role in the foreign policy agenda of the region. This is why Latin American governments are proposing the generation of SD schemes based on the knowledge and experience acquired by the organized diasporas. For example, Colombia is currently developing its SD strategy to include a better articulation of the scientific diaspora (Gual, 2020). To generate synergies in the aforementioned context, it is advisable for national scientists to articulate with networks of scientists abroad promoting “brain linkage.” The greatest challenge is to achieve this interdisciplinary and trans disciplinary linkage between actors and policies of science and diplomacy (Gual, 2020).

Today's globalized society is supported by a State that promotes public policies and an international bureaucracy that is emerging as the new instance of power. The globalized society is immersed in a world where all individuals can communicate and cooperate, whether they are in the West, in the East, in the North or in the South (Gómez Lee, 2008). The optimal situation to promote exchange with the diaspora in today's society is for governments to create conditions conducive to their existence, facilitating cooperation and access to dialogue. The role of facilitator is itself a form of network, with the government being the one who would be drawn into engagement with the diaspora, fostering capacity building, and mutual understanding (Aikins and White, 2011). From this perspective, it is necessary for Latin American governments to work on policies that integrate SD schemes and cooperation with the diasporas. Policies that are not only isolated events but become true SD strategies in the country's foreign policy.

## Methods

### General Objective of the Study

To analyze the interactions and initiatives identified between the organized scientific diaspora and the governments of their countries of origin in relation to SD processes, based on an initial review of existing organized (scientific) diaspora networks in the region, as well as on three representative cases from LAC, in order to provide recommendations and proposals for public policy to improve the interaction between the diaspora and their countries of origin.

### Design and Procedure

The present study is descriptive and exploratory. It follows a mixed research design. The main use of mixed methods in research is to strengthen the validity of the studies and the consistency of the resulting conclusions (Schoonenboom and Johnson, 2017). In addition, the combination of quantitative and qualitative methodologies contributes to a broader understanding of complex research problems (Creswell, 2009).

In the first part of the study, a survey was distributed to Latin American diaspora organizations in order to identify which of these groups were engaged in SD activities. In the second part, three Latin American scientific diaspora organizations were identified using as criteria their engagement in SD activities and their willingness and availability to participate in this study. These three diaspora groups participated in focus groups to understand their interactions with their home countries in SD activities.

Data collection was carried out between June and October 2021.

### Participants

#### Selection of Latin American and Caribbean Scientific Diaspora Organizations

The authors identified 27 Latin American and Caribbean scientific diaspora organizations based mostly in Europe and North America through online searching and the network's contact information. Organizations from individual or multiple countries were considered. All organizations selected had their own website or an official government page (save for Red de Uruguayos Universitarios en Finlandia-URUFI-) to ensure the organizations had a certain level of maturity. The scientific diaspora organizations selected also needed to be active and to have a highly-qualified focus or field. An attempt was made to include organizations across the region.

A quantitative questionnaire was created to determine the objectives of the selected diaspora organizations, and to identify SD projects, initiatives and interactions undertaken by these organizations. The questionnaire was divided into three sections: (i) sociodemographic analysis, (ii) objectives of the organization, (iii) characterization of SD activities.

Table 2 contains the 27 organizations identified and approached to complete the quantitative questionnaire. Among these, only 10 answered. Based on their responses, three were selected for a qualitative study through focus groups. The criteria for selection to the qualitative study included at least three of the following: (1) multidisciplinary focus, (2) engagement with homeland institutions, (3) residence of all members outside of their home country, (4) structured organization, (5) SD activities, (6) representation of different Latin American and Caribbean subregions.

**Table 2:** List of Latin American and Caribbean scientific diaspora organizations contacted.

**Name of organization**	**Country(ies) of origin**	**Region/Country of residence**	**Website**
Red de Investigadores Chilenos en Alemania (INVECA)	CL	DE	redinveca.cl
Investigadores Chilenos en Suiza (ICES)	CL	CH	ices-net.ch
Red de Investigadores Chilenos en Países Bajos (IN.NL)	CL	NL	in-nl.net
Nexos	CL	USA	nexoschileusa.org
Red de Investigación Chile-Canadá (REDICEC)	CL	CAN	redicec.com
UQCHILE	CL	QUEENSLAND, AUS	uqchile.com
Red de Investigadores Chilenos en España (Red INCHE)	CL	ES	twitter.com/redinche
Red de Científicas/os Argentinos en Alemania (RCAA)	AR	DE	rcae.info/category/alemania
Red de Científicas/os Argentinas/os en Israel	AR	IL	argentina.gob.ar/ciencia/raices/redes-exterior/israel
Red de Científicas/os Argentinas/os en Italia	AR	IT	rcai.it
Red de Científicas/os Argentinas/os en Países Bajos	AR	NL	sites.google.com/view/rcapb/inicio?authuser=1
Red de Científicas/os Argentinas/os en Reino Unido	AR	UK	argentina.gob.ar/ciencia/raices/redes-exterior/reino-unido
Red de Científicas/os Argentinas/os en Suiza	AR	CH	argentina.gob.ar/redes-de-cientificasos-argentinasos-en-el-exterior/en-suiza
Diáspora Brasileira de Ciência e Inovação na Alemanha	BR	DE	diaspora-ctibr.de
Fundación México-Estados Unidos para la Ciencia (FUMEC)	MX	USA	fumec.org
Centro Virtual de Altos Estudios de Altas Energias (CEVALE2VE)	VE, CO, PE, MX	Global	cevale2ve.org/es/inicio/
Diáspora Científica por Venezuela	VE	Global	sites.google.com/view/diplomaciacientificave
HIPATIA	CR	Global	hipatia.cr/dashboard/diaspora-cientifica
Caribbean Diaspora for Science Technology and Innovation-New England	Caribbean	NE-USA	cadsti-ne.org
Caribbean Diaspora for Science, Technology and Innovation (UK)	Caribbean	UK	cadsti.org.uk
Caribbean Diaspora for Science Technology and Innovation-Silicon Valley	Caribbean	SValley-USA	cadsti.org/cadsti-sv
Asociación Colombiana de Investigadores en Suiza (ACIS)	COL	CH	acis.ch
Asociación de Científicos Peruanos, Cientificos.pe	PE	Global	Cientificos.pe
Sinapsis	PE	Europa	sinapsis-peru.org
Research Experience for Peruvian Undergraduates	PE	USA	repuprogram.org
Universidad Simon Bolivar Alumni Association of America (USB alumni)	VE	Global	alumnusb.org/about-our-work
Red de Uruguayos Universitarios en Finlandia (URUFI)	UY	FI	uyredesuyredes.wordpress.com/redesuy-finlandia

It was not possible to know how many members partake in each one of the 27 organizations. Only the three interviewed organizations provided that information. The 10 organizations that filled in the questionnaire are a good representation of the reality of the study population as they include most of the subregions within LAC.

### Diaspora Organizations Participating in Focus Groups

Considering the criteria of willingness, availability and the organization of SD activities, the diaspora organizations URUFI, INVECA, and CEVALE2VE were chosen to participate in this study. INVECA has 120 members, 30 of them were active at the moment of conducting this research. URUFI has 22 members whilst CEVALE2VE has 20 active members. A focus group was conducted with each organization. Each focus group lasted approximately one and a half hours. The Interview Guide for the focus groups contained questions related to general information about the diaspora organization, the interviewees' conception of SD, the activities carried out by the diaspora with the governments of their countries of origin, and recommendations for improving the interaction between the diaspora and the governments of their country of origin. The persons interviewed at each session of the focus groups were either members of the board of directors or other leading members of the organizations, corresponding to four persons in the case of CEVALE2VE, three participants in the focus group of INVECA and two persons in the case of URUFI.

The following is a brief description of each of the diaspora organizations, their date of creation and the number of active members to date.

#### INVECA

The Network of Chilean Researchers in Germany (Red INVECA e.V.) started its activities in 2012. Since then, the network has brought together more than 120 Chilean researchers from different disciplines living in Germany to discuss academic issues, science policies and aspects related to the development of science, based on their own experiences. To generate these spaces for dialogue, several annual conferences have been held (Berlin, 2012; Heidelberg, 2013; Bamberg, 2014; Frankfurt, 2015; Berlin, 2016; Hamburg, 2017; Karlsruhe, 2018; Freiberg, 2019) that have allowed strengthening relationships with academic and scientific organizations in Chile and Germany, including official entities of both countries. Currently 30 members have active membership. Since 2015 the network has had legal status (RedINVECA, 2021).

#### URUFI

The Network of Uruguayan Academics in Finland (URUFI, by its Spanish acronym) started running regular meetings with its members in November 2016. It currently has 22 members with different profiles, some dedicated to the academic and scientific field and others to the business field. URUFI's mission is to be a tool for the linkage and mutual cooperation between Uruguayans in academic and specialized diaspora residing in Finland, in order to facilitate the creation of professional links and promote initiatives that stimulate the creation of joint projects (UYREDES, 2021).

#### CEVALE2VE

Centro Virtual de Altos Estudios de Altas Energías (CEVALE2VE) is an initiative of the Venezuelan scientific diaspora to strengthen academic training, networking, research and outreach in the area of High Energy Physics (HEP). Initially created to reach the Venezuelan academic community, it has now expanded to several Latin American countries. Activities began in 2014. Currently the activities of the network are mainly focused on the Latin American Alliance for Capacity Building in Advanced Physics (LA-CoNGA)^1^, a capacity building project in advanced physics co-funded by the ERASMUS+ program of the European Commission. LA-CoNGA physics has designed and developed an educational program within the framework of a Latin American Alliance in Advanced Physics for students in Colombia, Ecuador, Peru, and Venezuela. At present this organization has approximately 20 active members (CEVALE2VE, 2021).

### Data Analysis

For the analysis of the data obtained from the focus groups, the qualitative content analysis method (Mayring, 2000) was applied. MAXQDA software^2^ was used to facilitate the inductive organization of categories. The following categories were created:

- Network features
- Conceptions of science diplomacy
- Engagement initiatives of the diaspora with their governments
- Recommendations for diaspora-government linkages

This was followed by the research report, which included quotations from the focus groups conducted.

## Results

### Quantitative Results

**Figure 1:**
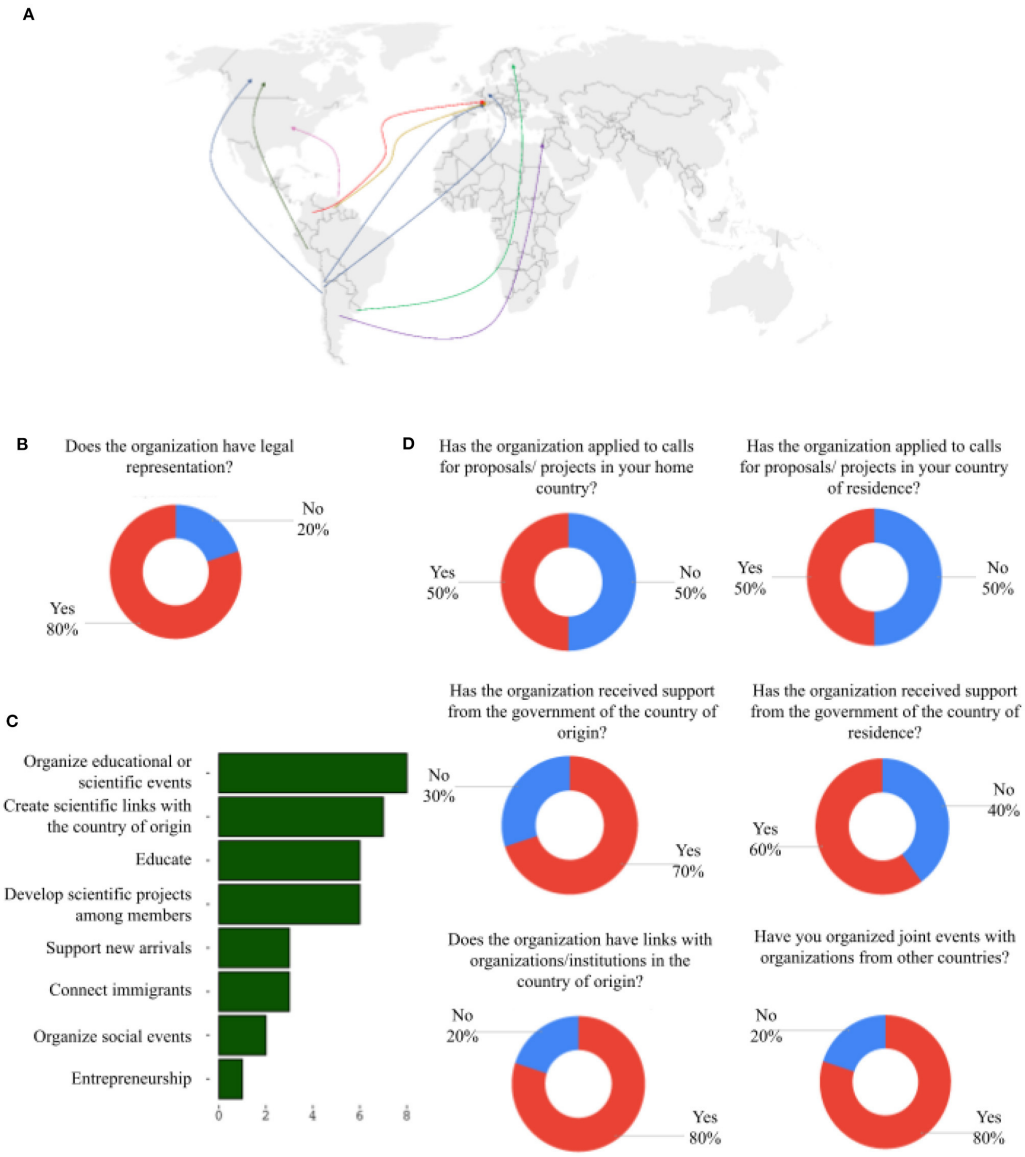
Analysis of diaspora networks. **(A)** The 10 countries of origin and countries of residence of the diaspora networks that answered the survey (see Table 2). **(B)** Proportion of diaspora networks with legal representation. **(C)** Frequency of objectives stated by the diaspora networks surveyed (*n* = 10). **(D)** Relationship of diaspora networks to their country of origin and their country of residence.

The diaspora organizations that answered the quantitative survey were mostly established in European and North American countries and originated from countries across South America and the Caribbean (Figure 1A and Table 2). Unfortunately, no diaspora organization from Central America participated in the survey. Most of the surveys were answered by the presidents or members of the governing boards of the organizations (80%), the majority of whom held a PhD (90%), and were working in either academia (70%) or the private sector (30%). Consistent with our focus on well-established organizations, 80% of organizations declared having legal representation (Figure 1B) and at least 50% had applied to calls for proposals or projects in their country of origin or country of residence (Figure 1D). The highly-qualified and scientific nature of these organizations was also evident in their objectives, which were mostly focused on educational and scientific goals, with other goals like connecting immigrants and organizing social events showing less prevalence (Figure 1C).

To understand the interactions and influence of these diaspora organizations, we sought to determine the types of past activities or relationships undertaken with government and other non-state actors. Seventy percent of the organizations reported receiving support from the government of the country of origin and 60% from the government of the country of residence (Figure 1D). In the case of the country of origin, this help was presented in the form of financial support, competitive funds or in-kind support for activities; use of embassy premises; support for event promotion; scholarships for students and participation of public institutions in events. Only embassies or foreign affairs offices were mentioned specifically as providers of support. In the case of the country of residence, support was reported in the form of financial support for events and projects (including projects in the country of origin), participation of public institutions in events and support for event promotion. The ministry of science, institutions for the integration of migrants, institutions that promote cooperation with Latin America and public universities were mentioned specifically as having provided support.

Around 80% of the diaspora organizations also noted having links to other entities in their respective country of origin (Figure 1D), which included universities, schools, research groups, associations, libraries, networks, national agencies for research, and embassies (of the country of residence in their country of origin). Events with organizations from other countries (i.e., not the country of origin or the country of residence) were also reported by 80% of participants (Figure 1D). Markedly, these events were mostly thematic scientific events (e.g., scientific congresses), with only two organizations reporting other themes like capitalization of scientific experiences or career opportunities.

**Figure 2:**
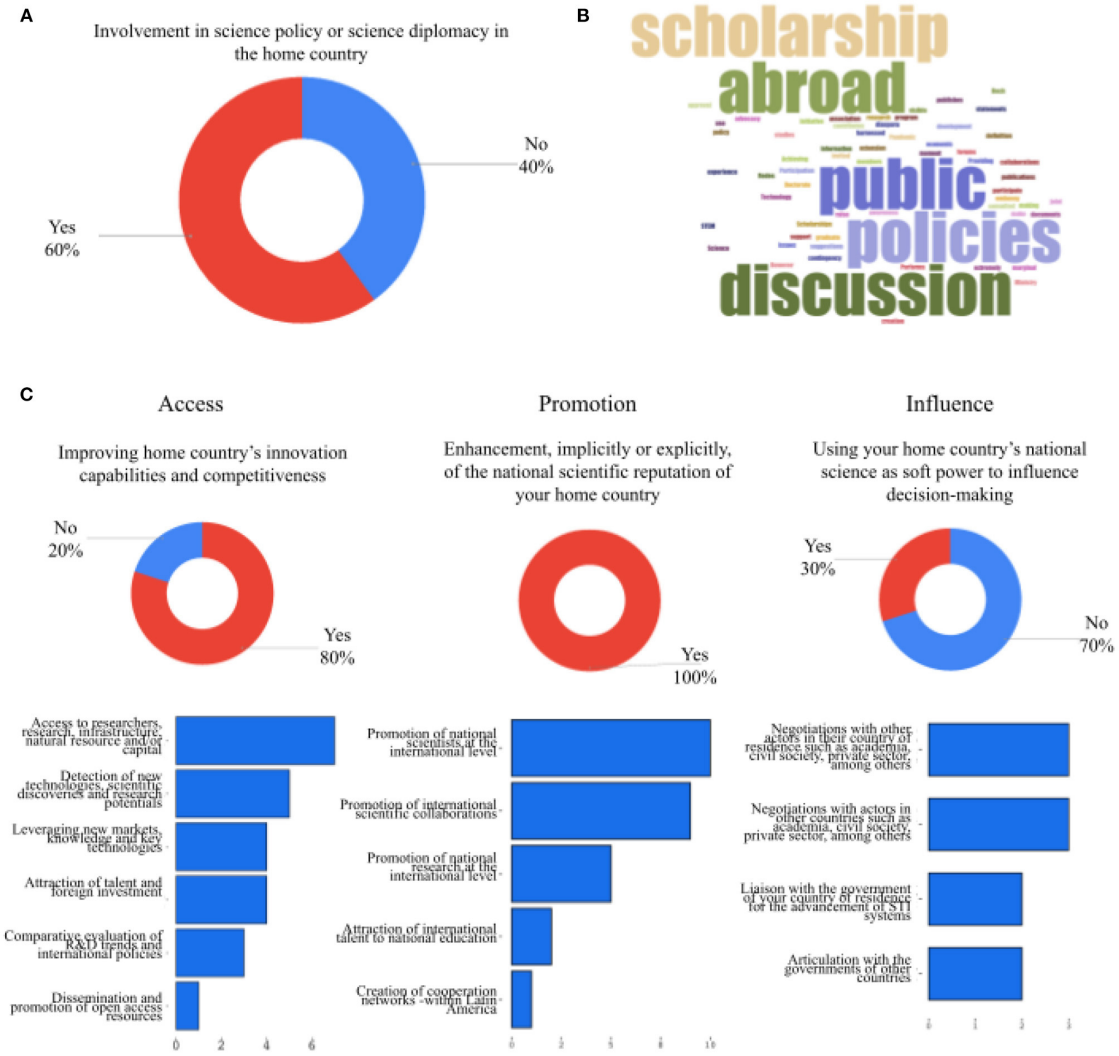
Science diplomacy engagement of the diaspora networks. **(A)** Proportion of diaspora networks that self-employed engaging in science diplomacy activities. **(B)** Word cloud of answers to the question of how these diaspora networks engage in science diplomacy. Generated at https://www.jasondavies.com/ from translated answers. **(C)** Science diplomacy activities undertaken by the diaspora networks based on the definition of science diplomacy by Flink and Schreiterer (2010). Created by Datawrapper.

In order to determine the engagement in SD practices, organizations were questioned explicitly on their participation in science policy or SD initiatives in their home country and implicitly through questions based on the “strategic approach” of SD by Flink and Schreiterer (2010). Sixty percent of organizations self-reported as being involved in science policy or SD initiatives (Figure 2A). Figure 2B contains a word cloud with the answers to the question of how these organizations had participated. These included direct contributions to public policy discussions, mediation with government for scholarship extensions, joint declarations with governments, publications, and collaborations, awareness of how science, technology, engineering and mathematics (STEM) can be harnessed for economic development and information visualization.

For the implicit approach, organizations were questioned about their activities and goals based on the three categories defined by Flink and Schreiterer (2010): Access, Promotion, and Influence (Figure 2C).

Regarding the type of SD activities reported:

- Access: 80% of the organization reported to be working toward improving their home country's innovation capabilities and competitiveness by creating access channels to researchers, research, infrastructure, new technologies and other resources.
- Promotion: 100% lead or participate in research and researchers' promotion activities, as well as network collaborations.
- Influence: 30% of the organizations communicate as one of their aims to use national science to influence decision-making in negotiations with actors in academia, civil society and the private sector outside their home country.

Together, these results indicate that highly-qualified diaspora organizations are active SD players, largely focused on promotion and access strategies.

### Qualitative Results

After conducting the focus groups, the following results are presented.

### INVECA

#### Network Features

This network stands out because of its governance and autonomy. It is an interdisciplinary and horizontal organization that uses democratic mechanisms for elections and has political autonomy. Its leaders serve in short 2-year periods and it builds on strategic alliances with key stakeholders in Germany. There is an important commitment and responsibility by each member, not only the leaders, to contribute to the network to help it develop.

Excerpts from interviews regarding network features:

*-From the beginning there is a continuity effect. The network was founded in 2012; also supported by the Argentinean diaspora; it started with a model and it grew. The board periods last 2 years and new people bring fresh ideas. We have also had public funding. The embassy gives us funds for our annual meeting. Each generation of the board has had important milestones; legal status, bank accounts, we have positioned the network with strategic partners. (Participant 1)*.

*-The autonomy of the network, political autonomy, which does not depend on the governments in power, the interdisciplinary nature of the network, the democratic mechanisms within the internal structure of the network, such as the leadership, with an expanded board where other members participate in relevant actions, and the strategic alliances with actors in Germany. This positioning in Germany has been very important. (Participant 3)*.

#### Conceptions of SD

The conceptions from this network refer to government-led activities involving researchers and the use of researchers for liaison with other countries. It includes the networking processes for science and facilitating scientific collaboration.

Excerpts from interviews regarding conceptions of SD:

*I have an academic bias with that, I understand it as international activities, directed or planned by governments where other scientific actors are involved in the relationship with other countries. But, when it is described as “diplomacy” I associate it with governmental activity. (Participant 3)*

*A little bit of what our network does; facilitating scientific collaboration, and providing interaction activities between different actors in the community. (Participant 1)*.

#### Engagement Initiatives of the Diaspora With Their Governments

The interviewees indicate some of the activities linking the diaspora with their governments, namely the international cooperation programs in various fields, or the strategic alliances with government entities. This network refers to funds to organize activities with the scientific diaspora and the strategies for outreach scientific activities.

Excerpts from interviews regarding initiatives of the diaspora with their governments:

*There are two forms of linkage; for example, the search for financing for network activities and generating strategic alliances with government entities, for example for the Chilean embassy in Germany. We have done some activities, for example, and we have been accompanied by Chilean government officials. (Participant 1)*

#### Recommendations for Diaspora-Government Linkages

Building networks between diaspora and government entities is one of the key recommendations from this set of interviewees. Diaspora should present their activities to their governments. They also mention that governments should generate public funds for diaspora activities in support of funding annual meetings of diaspora networks. INVECA suggests the government-to-government transfer of good practices on diasporas.

Excerpts from interviews regarding diaspora-government linkages:

*Positioning oneself vis-à-vis the government, presenting projects, activities; in our case, such as the annual meeting is a scientific activity, which has a return by allowing Chile to present itself in another country, in this case Germany, given that this is a public activity. This is an example of positioning. (Participant 2)*

INVECA's results are presented below in Table 3, divided into the aforementioned categories.

**Table 3:** INVECA's results.

Network features	Political autonomy
	Leadership in short 2-year periods
	Horizontal network
	Use of democratic mechanisms for elections
	Strategic alliances with stakeholders in Germany
	Interdisciplinary
Conceptions of science diplomacy	Government-led activities involving researchers
	Facilitating scientific collaboration
	Networking processes for science
	Use of researchers for liaison with other countries
Recommendations for diaspora-government linkages	Diaspora should present their activities to their governments
	Funding annual meetings of diaspora networks
	Generating public funds for diaspora activities
	Building networks between diaspora and government entities
	Government-to-government transfer of good practices on diasporas
Engagement initiatives of the diaspora with their governments	Funds to organize activities with the scientific diaspora
	Strategies for the outreach of scientific activities
	International cooperation programs in various fields
	Building strategic alliances with government entities

### URUFI

#### Network Features

Despite URUFI having no legal status, it is a compact group with a shifting population and a defined mission and vision. It is officially constituted and network members consider it as an instrument of linkage and cooperation between Uruguayan university students, professionals and academicians in Finland.

Excerpts from interviews regarding network features:

*URUFI does not have legal status and we are not officially linked to the government. What we have in practice is that the Uruguayan Embassy in Helsinki invites us to events and meetings, but it is not official.(…)The good thing is that we are a small, compact group and that we communicate very well. (Participant 4)*

*We have a mission, a vision, although we had some identity crisis at some point. We seek to be a group that is an instrument of linkage and cooperation between Uruguayan university students in Finland and to include all types of academics: university students, professionals and scholars. The goal is to include everyone. (…) URUFI has almost 5 years of validity. Our grouping is very mobile. (Participant 5)*

#### Conceptions of SD

For URUFI the concept of SD has many meanings and there is no single definition. It can be defined as an interconnection between science, authorities' decisions—making positions, academicians, researchers, and networks, where ideas and projects are exchanged to improve the world.

Excerpts from interviews regarding conceptions of SD:

*It is about relationships between universities, scientists and networks where ideas and projects could be exchanged to improve the world through this; it is a synergy. (Participant 5)*

*The concept has many meanings, there is no single definition…it is an articulation between science, the transmission of science in the world, but also another component of people who are in places where they can make decisions, such as politicians and authorities. (Participant 4)*

#### Engagement Initiatives of the Diaspora With Their Governments

There are several initiatives led by URUFI but not defined as SD strategies. For example, there are alliances for research funding by ANII^3^, the *Agencia Nacional de Investigación e Innovación* from Uruguay, or by “Uruguay XXI,” an agency which is heavily involved in achieving the Sustainable Development Goals (SDGs). URUFI has also established contact with the Uruguayan Foreign Ministry for the participation in an event called “Active Citizenship for Development” which brought together qualified Uruguayan diaspora around the world in Montevideo.

Excerpts from interviews regarding diaspora engagement with their governments:

*I can think of ANII, the National Agency for Research and Innovation. This organization has had many initiatives to finance research, especially in the area of doctoral studies, issues related to entrepreneurship (…). (Participant 4)*

*Events such as meetings, gatherings and others are held, in which we are invited by universities, the Uruguayan Foreign Ministry, among others. (Participant 5)*

#### Recommendations for Diaspora-Government Linkages

URUFI believes that actions should be carried out organically and developed before being presented to governments for support or funding. URUFI thinks that it is possible to create a “Network of networks,” to generate links and build bridges with the authorities of the Ministry of Foreign Affairs of Uruguay.

Excerpts from interviews regarding diaspora-government linkages:

*In my personal opinion, a few years ago we contacted ANII and the Ministry of Foreign Affairs and at that time I do not know why we were unable to materialize the ideas we were putting forward, for example the “Network of networks” initiative (…). Then I thought we could do it the other way around. Instead of first going to the authorities to move an initiative forward, we first had to establish those links ourselves, such as the creation of the “Network of networks.” We were in contact with an Uruguayan researcher in Barcelona with whom we have generated initiatives, ideas and a very fruitful connection and we also agreed with the idea that we must first create the links and then go to the authorities, because otherwise we might not be able to inspire enthusiasm in the governmental authorities, and this way we would do it more organically. (Participant 4)*

URUFI's results are presented below in Table 4, divided into the aforementioned categories.

**Table 4:** URUFI's results.

Network features	It has no legal status
	It is not officially constituted
	It operates physically at the invitation of the Uruguayan Embassy in Helsinki.
	Small and compact group
	Mobile grouping with 5 years of validity
	Clear identity of Uruguayan university students in Finland
	Defined mission and vision
	Instrument of linkage and cooperation between Uruguayan university students in Finland
Conceptions of science diplomacy	Relationships between academicians, researches and between networks where ideas and projects could be exchanged to improve the world through this
	It is an interconnection between science and people in decision-making positions, such as politicians and authorities.
Recommendations for diaspora-government linkages	Contact with offices of the Ministry of Foreign Affairs
	Creating a network of networks
	Generate links and then communicate with the authorities
Engagement initiatives of the diaspora with their governments	Research funding initiatives at ANII, the National Agency for Research and Innovation

### CEVALE2VE

#### Network Features

CEVALE2VE was originally composed of Venezuelans in diaspora, but nowadays, it includes people of different nationalities who have a sense of identity and belonging to the network. Many of its members were trained in the same disciplines of knowledge (physics), this is why there is a previous link between them. CEVALE2VE has strength in fundraising, which has greatly contributed to the educational and outreach work of the network, especially with physics students in Latin America.

Excerpts from interviews regarding network features:

*Initially we created CEVALE with only Venezuelans, but then we brought in people of other nationalities. Our idea is very simple: many of us did the same physics: particle physics. (Participant 6)*

*There were a number of funding sources. We learned how to apply for international projects (…). We created funding sources so that our students could do international internships. (Participant 7)*

#### Conceptions of SD

One of the definitions provided by CEVALE2VE members indicates the use of the scientific role to open doors with decision makers in the political arena. They also stress the importance of collaboration between countries to strengthen STI and the creation of international networks for researchers. Emphasis is also placed on the role of the scholar as a diplomat, establishing alliances, and collaborations for science.

Excerpts from interviews regarding conceptions of SD:

*It sounds to me like taking advantage of the doctoral degree we have to get closer to those who make public policy. (Participant 6)*

*Networking, building knowledge, and establishing relationships. We have always had colleagues coming and going. If you imagine SD from the frontiers, it's a rather natural process. (…) SD changes from its context; building collaborations and knowledge between different cultures. (Participant 7)*

#### Engagement Initiatives of the Diaspora With Their Governments

The interviewees indicated that through government funded doctoral scholarship programs they have been able to get training abroad and some of them have returned to their country of origin. This allowed them to participate in high-level training and to strengthen the relationship with countries that have good international links. Given the political situation in Venezuela, CEVALE2VE's relationship with its country of origin has been mainly with universities.

Excerpts from interviews regarding initiatives of the diaspora with their governments:

*(…) Many people were trained and came back with those scholarships such as BECAS FUNDACIÓN GRAN MARISCAL DE AYACUCHO; the most important thing is that it helped to generate connections. (Participant 9)*.

*There have always been scholarship systems to interact with countries, which have developed very good relations. (…) Now we have an Erasmus project where there are eight universities from Latin America. This scalability made it possible to bring in other people and countries. High-performance physicists are also accustomed to tribalism. To be together. (Participant 6)*

#### Recommendations for Diaspora-Government Linkages

CEVALE2VE recommends removing the stigmas related to the diaspora, recognizing its value and the actions it has been developing with and in other cases without the support of the government. They also highlight the potential of creating the conditions to work in and for the country, promoting remote actions to enhance STI through virtual means. On the other hand, they consider open science as an engine for the collaboration between countries; they also mention the contribution of the diaspora to generate connections outside their country of origin, which can be of great importance for the development of STI systems.

Excerpts from interviews regarding diaspora-government linkages:

*You have to start by talking; get to know the diaspora and see what they are doing (…); you have to start with dialogue. (Participant 8)*

*Venezuela has a very special scenario. The first thing is to create conditions. You are trained abroad and there is no place where you can work or have a decent salary. It is important not to see the individual who is abroad as someone who failed and left everything behind; with virtuality you can establish meaningful connections with scientists. (Participant 9)*

CEVALE2VE's results are presented below in Table 5, divided into the aforementioned categories.

**Table 5:** CEVALE2VE's results.

Network features	Giving back to the country
	Identity and sense of belonging
	Previous connections among participants
	Collaboration with diasporas of diverse nationalities
Conceptions of science diplomacy	Collaboration between countries for STI
	Creating international networks for researchers
	Contact between researchers and public policy
Recommendations for diaspora-government linkages	Encouraging the establishment of consortia for STI
	Creating conditions to work in and for the country
	Engaging politicians with researchers
	Fostering remote actions on STI
	Diagnosis of the diaspora and the actions it carries out
	Open science as an assembling engine among countries
	Links with the diaspora through the universities
	Removing stigmata related to diaspora

## Discussion

Scientific diaspora organizations are primarily connected to SD as evidenced in the results, from an approach of “Access” (Flink and Schreiterer, 2010) to laboratories, training, capacities, good practices in STI, and also from the dimension of “Promotion” (Flink and Schreiterer, 2010), by promoting science and training carried out in the country of origin. As evidenced in the previous section, for the scientific diaspora organizations interviewed, SD is mainly about connecting researchers, creating alliances between countries to promote STI and using science to support decision makers. In this sense, from the perspective of scientific diaspora organizations, SD is about establishing bridges between their country of origin and the host country to advance science. However, this conception is not clear to governments, since there is a lack of consistent and sustainable policies and programs to involve the organized scientific diaspora, as has been shown throughout this article.

Scientific diaspora organizations seek to support national interests, but also those of regional and global nature (Gluckman et al., 2017), mainly through significant scientific alliances. However, one of the most present manifestations in this study is that the diaspora actively collaborates with the capacity building of personnel in their country of origin (Arunachalam et al., 2017), but also the important support provided by the diaspora for individual researchers or networks in their international scientific engagement process. Definitely one of the outcomes observed in this study is the support carried out by the scientific diaspora for the establishment of scientific collaborations and international cooperation projects, where it can contribute to remove barriers, both cultural, scientific and technical, with counterparts, especially those located in countries of the Global North.

Something present in diaspora organizations is the interest in giving something back to the country of origin, especially in terms of capacity building, but also in terms of networking and outreach. As governments have not been consistent in their relationship with the scientific diaspora, these organizations connect with non-governmental actors, as seen in the case of CEVALE2VE, who have an important involvement with higher education institutions.

According to López-Vergés et al. (2021), there are no consistent channels of communication with the scientific diaspora, and many of the public policies are not directed to organized diaspora. This is troublesome, because as we observed in the results, the organized diaspora mainly wants to seek an effective and long-term engagement with their country of origin, but from outside those countries. As observed in the results, a generalized problem in this region is the lack of quality working conditions that allow the recruitment of highly qualified diaspora; this has caused that policies oriented to “brain circulation” have not been successful in the region in the long term. In this sense, the “brain linkage” approach proposed by Tejada et al. (2013) is the most effective for LAC and other emerging economies, as it takes advantage of the potential of the diaspora in terms of networking, connections, knowledge transfer and support for public policy and even the development of innovation in the business sector. Moreover, support to the organized diaspora can be enhanced so that it can advance and become a diaspora network, with actors from the country of origin as well as the host country and even others, favoring the flow of knowledge supported by virtuality (Grossman, 2010), as observed in the case of CEVALE2VE.

Based on the review carried out, networking, scientific collaboration and joint projects are the foundation of scientific diaspora networks. Providing scientific advice and forging links with the governments of their countries of origin are only indirect or utilitarian (financial) purposes. The evident benefits of scientific diaspora organizations are related to the support among network members. The use of virtual tools is a practical approach to link the scientific diaspora with national issues and facilitate knowledge exchange.

## Recommendations and Policy Implications for Diaspora Organizations and its Governments

Based on the evidence provided by the diaspora organizations participating in this research, a set of recommendations for diaspora organizations and the national governments interested in strengthening the interaction with the diaspora are presented in this section. This set is within the schemes of SD and it could strengthen some of the still under construction policies for SD in LAC.

Recommendations for the welfare of the diaspora organizations have been divided in seven categories:

- Mission and vision: defining a clear mission, vision, and organization values is important to establish the identity of the organization and generate a sense of belonging in their members. Clear definition of the role of each member inside the organization. Flexibility, as in many cases the participation in the organization is done in their free time. Recognition of the work of each member.
- Governance: create horizontal, non-partisan, democratic, and rotational governance to ensure active participation in development and decision-making of the organization. To ensure independence from governments, it is crucial not only to avoid partisan statements when discussing science policy but also to secure third party funding.
- Education: competences and needs of the members of the diaspora organizations should be assessed in relation with SD. This will enable the identification of suitable training on SD for the organization.
- Collaboration/relationships: strengthen the collaboration with origin and home countries governments, academic institutions, embassies and other diaspora organizations to exchange best practices. Training on SD can be targeted and more effective in terms of alliance with government and public policy development.
- Communication: outreach activities to communicate objectives and results from the diaspora organization, making the organization findable. Make use of remote/online communication tools for strengthening relationships with home countries and promote social interaction.
- Open use of resources: ensure transparent accessibility to organization resources and reusability of resources through coordinated efforts.
- Sustainability: search for funding, links with governments and other third party organizations (see relationships).

As for the governments willing to further enhance the scientific diaspora in line with the brain linkage approach, we propose the following recommendations and policy implications based upon the results of this research:

- Count: generate an inventory or diagnosis of a country's scientific diaspora and their actions.
- Create: fund calls to support the activities of the diaspora organizations and or collaborations between national and diaspora organizations. Improvement of conditions to work in and for the country.
- Collaborate: Involve the scientific diaspora organization to contribute to scientific policy development, strategies on SD, events, activities, etc. Collaboration programs between national and diaspora communities at the academic and government level. This collaboration should not compromise the independence of the scientific diaspora organizations, they should retain their ability to operate in a manner that is insulated from the influence of political actors.
- Recognize: recognition of the role of the diaspora in the scientific development of the countries.
- Share: transferring good practices from government to government related to scientific diasporas. Promoting open science as an assembling engine among countries.

## Conclusions

Diaspora organizations from LAC engage with government and non-state actors and are active SD players by promoting the scientific developments of their country as well as by enabling access to research resources through links between their country of origin and their country of residence. These efforts are mostly done implicitly without a clear SD strategy. Indeed, the study confirmed that the definition of SD remains vague and ambiguous in these groups. Nevertheless, these diaspora organizations have complex structures, networks and projects that have benefited their country of origin as well as their diaspora members.

In cases where there are not enough diaspora networks related to a given country, a regional one could be generated or the creation of a network of networks could be encouraged, enhancing diaspora development in the framework of regional blocs. The lack of economic resources and political strategies implies that science still plays a secondary role, STI policies have been adjusted to the political-economic reality of each country, characterized by significant periods of uncertainty and instability. Government plans and public policies should include provisions to support qualified diaspora organizations working on an internationalization strategy for STI, facilitating spaces for diaspora participation in STI ministries, as well as government agencies and the Ministry of Foreign Affairs.

The LAC region as seen in the qualitative and quantitative data of the study is a clear reflection that the interest to maintain links with the country of origin does not emanate from the States, which leads us to reflect on the need as a region to establish public policies that allow sustainability in the collaborations with the States. As mentioned before, for the organized scientific diaspora SD is about creating bridges between the host and home country to advance STI. We need a more homogeneous and balanced growth between the scientists who return to their countries of origin and those who settle in the host countries, and rather than a loss of talent, guarantee gain and exchange of opportunities, such as those presented in the recommendations.

## Data Availability Statement

The raw data supporting the conclusions of this article will be made available by the authors, without undue reservation.

## Author Contributions

All authors listed have made a substantial, direct, and intellectual contribution to the work and approved it for publication.

## Conflict of Interest

The authors declare that the research was conducted in the absence of any commercial or financial relationships that could be construed as a potential conflict of interest.

## Publisher's Note

All claims expressed in this article are solely those of the authors and do not necessarily represent those of their affiliated organizations, or those of the publisher, the editors and the reviewers. Any product that may be evaluated in this article, or claim that may be made by its manufacturer, is not guaranteed or endorsed by the publisher.

## Footnotes

^1^ https://laconga.redclara.net/

^2^ https://es.maxqda.com/

^3^ https://www.anii.org.uy

## References

[B1] Aikins K. White N. (2011). Diaspora Matters: Global Diaspora Strategies Toolkit. Dublin: The Networking Institute.

[B2] Arunachalam R. Gupta R. Relia S. (2017). Better diplomacy and better science for better development: a way forward fulfilling post-201 development agenda and sustainable development goals, in S and T Diplomacy and Sustainable Development in the Developing Countries, eds Miremadi T. Arabzai A. Relia S. (New Delhi: Daya Publishing House), 65–78.

[B3] Bonilla K. Serafim M. Bámaca-López E. (2021). Science diplomacy in ecuador: political discourse and practices between 2007 and 2017. Front. Res. Metr. Anal. 6:656969. 10.3389/frma,.2021.656969 34046544 PMC8144718

[B4] Cancillería de Colombia. (2022a) Colombia Nos Une. Available online at: http://www.colombianosune.com/ (accessed February 5, 2022).

[B5] Cancillería de Colombia. (2022b) El Plan Retorno Positivo del Ministerio de Relaciones Exteriores ha Beneficiado a 3.723 Colombianos que Regresan al País Colombia Nos Une. Available online at: http://www.colombianosune.com/noticia/PlanRetornohaBeneficiado3723Colombianos (accessed February 5, 2022).

[B6] Cancillería de Colombia. (2022c) Registro Para Acogerse a Ley 1565 de 2012 Colombia Nos Une. Available online at: https://www.colombianosune.com/ejes/acompa%C3%B1amiento-al-retorno/rur (accessed February 5, 2022).

[B7] CEVALE2VE (2021). Qué es CEVALE2VE? Available online at: http://www.cevale2ve.org/es/inicio/ (Accessed December 16, 2021).

[B8] Chaparro F. Jaramillo H. Quintero V. (2004). Aprovechamiento de la Diáspora e Inserción en Redes Globales de Conocimiento: El Caso de la Red Caldas. Bogotá: Banco Mundial.

[B9] Chiriboga Salazar M. A. (2019). Análisis de la Implementación del Proyecto Prometeo Sobre las Publicaciones de las Instituciones de Educación Superior en el Ecuador Para el Período 2011-2017. Repositorio Digital USFQ. Available online at: http://repositorio.usfq.edu.ec/handle/23000/8258

[B10] Comunidad y Mercado Común del Caribe (1973). Tratado De Chaguaramas Revisado Por El Que Se Establece La Comunidad Del Caribe Con Inclusión Del Mercado Único Y La Economía De La Caricom Available online at: https://caricom.org/documents/11109-treaty_caricom_2-spanish.pdf

[B11] CONCYTEC (2020). Encuentro Científico Internacional 2020 Reunirá a más de 75 Científicos Peruanos y Extranjeros. Available online at: https://portal.concytec.gob.pe/index.php/noticias/2308-encuentro-cientifico-internacional-2020-reunira-a-mas-de-75-cientificos-peruanos-y-extranjeros (accessed July 24, 2020).

[B12] Congreso de la República Colombiana (2021). Ley 2136 de 2021. Available online at: https://dapre.presidencia.gov.co/normativa/normativa/LEY%202136%20DEL%204%20DE%20AGOSTO%20DE%202021.pdf (accessed August 4, 2021)

[B13] Congreso de la República Peruana (2013). LEY No 30001. Availble online at: https://leyes.congreso.gob.pe/Documentos/Leyes/30001.pdf (accessed March 14, 2013).

[B14] Creswell J. (2009). Research Design. Qualitative, Quantitative, and Mixed Methods Approaches. Thousands Oaks, CA: SAGE Publications.

[B15] Diaspora for Development (2021). Diaspora Engagement Map. Available online at: https://diasporafordevelopment.eu/diaspora-engagement-map/ (accessed June 28, 2021).

[B16] Diaspora Instituut Suriname. (2022). Available online at: https://diaspora.sr/en/homepage/ (accessed February 6, 2022).

[B17] Echeverría King L. F. González D. A. Andrade-Sastoque E. (2021). Science diplomacy in emerging economies: a phenomenological analysis of the colombian case. Front. Res. Metr. Anal. 6:636538. 10.3389/frma.2021.636538 33997599 PMC8120157

[B18] Echeverría L. Aquino K. Widmaier C. (2020). Science diplomacy and sustainable development goals: a latin american perspective. Sci. Diplom. Rev. 2, 3–13. 10.21500/23825014.4570

[B19] ECI. (2022). International Scientific Meeting (ECI), since 1993. Available online at: https://eciperu.net/category/noticias/ (accessed February 6, 2022).

[B20] Epping E. (2020). Lifting the smokescreen of science diplomacy: comparing the political instrumentation of science and innovation centres. Human. Soc. Sci. Commun. 7:111. 10.1057/s41599-020-00599-4

[B21] Epstein G. S. Heizler O. (2016). The Formation of Networks in the Diaspora. IZA Discussion Papers No. 9762. Bonn: Institute for the Study of Labor (IZA). Available online at: https://www.econstor.eu/bitstream/10419/141521/1/dp9762.pdf

[B22] Fernández M. (2008). Diáspora: la complejidad de un término. Rev. Venezolana Anál. Coyunt. 15, 305–326.

[B23] Flink T. Schreiterer U. (2010). Science diplomacy at the intersection of SandT policies and foreign affairs: toward a typology of national approaches. Sci. Publ. Policy 37, 665–677. 10.3152/030234210X12778118264530

[B24] Gëdeshi I. King R. (2019). The Albanian scientific diaspora: can the brain drain be reversed?. Migrat. Dev. 1:23. 10.1080/21632324.2019.1677072

[B25] Gluckman P. D. Turekian V. Grimes R. W. Kishi T. (2017). Science diplomacy: a pragmatic perspective from the inside. Sci. Diplom. 6:4.

[B26] Gómez Lee I. (2008). Las políticas públicas en la sociedad globalizada, in Observatorio de Políticas Públicas de la Facultad de Finanzas, Gobierno y Relaciones Internacionales, ed Opera (Bogotá: Universidad Externado de Colombia), 175–195.

[B27] Grossman M. (2010). Diaspora Knowledge Flows in the Global Economy. Management Faculty Publications. Available online at: https://vc.bridgew.edu/management_fac/18 (accessed November 15, 2021).

[B28] Gual M. (2020). Diplomacia Científica en América Latina y el Caribe. Estrategias, Mecanismos y Perspectivas Para Fortalecer la Diplomacia de la Ciencia, Tecnología e Innovación. Available online at: http://forocilac.org/wp-content/uploads/2020/11/PolicyPapers-DiplomaciaCientifica-ES.pdf (accessed May 24, 2021).

[B29] Guellec D. Cervantes M. (2002). International mobility of highly skilled workers: from statistical analysis to policy formulation” in International Mobility of the Highly Skilled, OECD 2001, 95. Available online at: https://read.oecd-ilibrary.org/employment/international-mobility-of-the-highly-skilled_9789264196087-en#page95

[B30] Hornsby D. Parshotam A. (2018). SD, epistemic communities, and practice in Sub-Saharan Africa. Glob. Policy 9, 29–34. 10.1111/1758-5899.12565

[B31] Instituto de los Mexicanos en el Exterior (2018). Red Global MX. https://www.gob.mx/ime/acciones-y-programas/red-global-mx

[B32] International Organization for Migration Migration Policy Institute (2012). Developing a Road Map for Engaging Diasporas in Development. Available online at: https://publications.iom.int/system/files/pdf/diaspora_handbook_en_for_web_28may2013.pdf (accessed February 12, 2022).

[B33] Jarquin-Solis M. Mauduit J. (2021). Institutional capacity for science diplomacy in Central America. Front. Res. Metr. Anal. 6:663827. 10.3389/frma.2021.663827 34337309 PMC8317690

[B34] López-Vergés S. Macías-Navarro L. Hernández-Mondragón A. C. Corrales-Aguila E. Gual Soler M. Guerra M. (2021). Closing the gap between emerging initiatives and integrated strategies to strengthen science diplomacy in Latin America. Front. Res. Metr. Anal. 6:16. 10.3389/frma.2021.664880 33912788 PMC8072213

[B35] Lujan Leiva M. (2005). La Emigración de Profesionales y las Políticas de Vinculación. Una Perspectiva Social - Histórica del Caso Argentino. Mar del Plata; V Coloquio Internacional sobre Gestión Universitaria en América del Sur.

[B36] Martinez Pizzaro J. (2005). Globalizados, pero Restringidos. Una Visión Latinoamericana del Mercado Global de Recursos Humanos Calificados. Santiago de Chile: CEPAL.

[B37] Mayring P. (2000). Qualitative content analysis. Forum Qualitative Sozialforschung/Forum Qual. Soc. Res. 1:2. 10.17169/fqs-1.2.1089

[B38] Meyer J. B. (2015). Diáspora: Hacia la Nueva Frontera. Available online at: https://horizon.documentation.ird.fr/exl-doc/pleins_textes/divers15-11/010065907.pdf (accessed November 7, 2021).

[B39] Ministério das Relações Exteriores (2017). Actions to Benefit Brazilians Abroad. Available online at: https://www.gov.br/mre/en/contact-us/press-area/press-releases/actions-to-benefit-brazilians-abroad (accessed June 26, 2017).

[B40] Ministerio de Ciencia Tecnología e Innovación (2021). Secretaría de Planeamiento y Políticas en Ciencia, Tecnología e Innovación. Red de Argentinos/as Investigadores/as y Científicos/as en el Exterior (RAICES). Available online at: https://www.argentina.gob.ar/ciencia/raices (accessed June 28, 2021).

[B41] Ministerio de Ciencia y Tecnología de Colombia (2014). Convocatoria es Tiempo de Volver 2014. Minciencias. Available online at: https://minciencias.gov.co/convocatorias/2014/convocatoria-es-tiempo-volver-2014 (accessed February 5, 2022).

[B42] Ministerio de Educación Ecuador. (2022) Franco Pombo, M. Acuerdo No 0019-13. Available online at: https://educacion.gob.ec/wp-content/uploads/downloads/2013/03/ACUERDO-0019-13.pdfhttps://educacion.gob.ec/wp-content/uploads/downloads/2013/03/ACUERDO-0019-13.pdf (accessed February 6, 2022).

[B43] Ministerio de Relaciones Exteriores de Chile (MRREE) (2021). Dirección Para las Comunidades Chilenas en el Exterior (DICOEX). Available online at: https://minrel.gob.cl/ministerio/direcciones/direccion-asuntos-consulares-y-de-inmigracion/dicoex%20https://chilesomostodos.gob.cl/ (accessed May 18, 2021).

[B44] Ministerio de Salud Pública. (2022a) Arranca Segunda Fase del Plan Ecuador Saludable… Vuelvo por Ti. Available online at: https://www.salud.gob.ec/plan-ecuador-saludable-vuelvo-por-ti/ (accessed February 5, 2022).

[B45] Ministerio de Salud Pública. (2022b) Plan “Ecuador Saludable Vuelvo por ti” Aumenta su Acogida Entre Profesionales de la Salud Nacionales y Extranjeros. Available online at: https://www.salud.gob.ec/plan-ecuador-saludable-vuelvo-por-ti-aumenta-su-acogida-entre-profesionales-de-la-salud-nacionales-y-extranjeros/ (accessed February 5, 2022).

[B46] Minto-Coy I. (2009). Diasporas and Development: An Assessment of the Irish Experience for the Caribbean. Available online at: https://caricom.org/conference-on-the-caribbean-2007-diaspora-forum-seen-as-major-highlight/

[B47] Organización Internacional para las Migraciones (2007). Diásporas Como Agentes Para el Desarrollo en América Latina y el Caribe. Available online at: https://www.iom.int/sites/g/files/tmzbdl486/files/jahia/webdav/site/myjahiasite/shared/shared/mainsite/media/docs/news/4diaspora_desarrollo.pdf (accessed November 12, 2021).

[B48] Plaza S. (2013). Diaspora resources and policies, in International Handbook on the Economics of Migration, eds Constant A. Zimmermann K. F. (Cheltenham; Northampton, MA: Elgar), 505–529.

[B49] Presidencia de la República Oriental del Uruguay, Secretaría de Prensa y Difusión. Programa de Vinculación de los Uruguayos Altamente Calificados Residentes en el Exterior. (2001) Available online at: http://archivo.presidencia.gub.uy/noticias/archivo/2001/junio/2001062606.htm (accessed June 26, 2001).

[B50] RedINVECA (2021). Historia. Available online at: https://www.redinveca.cl/historia/ (accessed December 16, 2021).

[B51] Rungius C.h. Flink T. Degelsegger-Márquez A. (2018). State-of-the-Art Report: Summarizing Literature on Science Diplomacy Cases and Concepts. Deliverable 2.2. Vienna: S4D4C. Available online at: https://www.s4d4c.eu/wp-content/uploads/2018/08/S4D4C_State-of-the-Art_Report_DZHW.pdf (accessed May 24, 2021).

[B52] Schoonenboom J. Johnson R. B. (2017). How to construct a mixed methods research design. Kolner Zeitsch. Soziol. Sozialpsychol. 69, 107–131. 10.1007/s11577-017-0454-1 28989188 PMC5602001

[B53] SENACYT (2012). Convocatoria Pública Continua de Captación de Talento Comprobado Para I+D (CAP). Available online at: https://www.senacyt.gob.pa/convocatorias/cerrada2012/descargas/convocatorias2012012/CAP2012/Anuncio%20Captacion%20de%20Talento%202012%20endoso%20Director%20I+D%20y%20OAL%20(FINAL).pdf (accessed February 6, 2022).

[B54] Shin G. W. Moon R. J. (2018). From brain drain to brain circulation and linkage, in Shorenstein Asia-Pacific Research Center Working Paper (Stanford, CA: Stanford University). Available online at: https://fsi-live.s3.us-west-1.amazonaws.com/s3fs-public/brain_drain_to_circulation_and_linkage_0.pdf (accessed January 2 2022).

[B55] Taks J. (2006). Migraciones internacionales en Uruguay: de pueblo trasplantado a diáspora vinculada. Theomai 14, 139–156.

[B56] Tejada G. (2007). Diásporas científicas. Una oportunidad para impulsar el desarrollo de México, in Serie Migración: Causas, Consecuencias y Recomendaciones, ed Universidad Iberoamericana (México: Programa de Asuntos Migratorios; Universidad Iberoamericana), 1–22. Available online at: https://infoscience.epfl.ch/record/146995/files/

[B57] Tejada G. Varzari V. Porcescu S. (2013). Scientific diasporas, transnationalism and home-country development: evidence from a study of skilled Moldovans abroad. Southeast Eur. Black Sea Stud. 13, 157–173. 10.1080/14683857.2013.789674

[B58] The Royal Society (2010). New Frontiers in Science Diplomacy: Navigating The Changing Balance of Power. Available online at: https://royalsociety.org/~/media/Royal_Society_Content/policy/publications/2010/4294969468.pdf (accessed July 1, 2021).

[B59] Thompson E. (2018). Science Diplomacy within sustainable development: a SIDS perspective. Glob. Policy. 9, 45–47. 10.1111/1758-5899.12515

[B60] Tigau C. (2010) Latin American scientific diaspora: before and after the economic recession (dissertation). Toronto, CA: Universidad Nacional Autónoma de México, Mexico City, Mexico.

[B61] Turekian V. (2018). The evolution of science diplomacy. Glob. Policy 9:3. 10.1111/1758-5899.12622

[B62] Uruguay Presidencia (2018). Ciudadanía Activa para el Desarrollo. Available online at: https://www.gub.uy/presidencia/comunicacion/audios/completos/ciudadania-activa-para-desarrollo (accessed June 28, 2021).

[B63] UYREDES (2021). URUFI Finlandia. Available online at: https://uyredesuyredes.wordpress.com/redesuy-finlandia/ (accessed December 16, 2021).

